# EARLY-LIFE STRESSORS, ADULT AFFECTIVE REACTIVITY TO DAILY STRESSORS, AND MORTALITY RISK

**DOI:** 10.1093/geroni/igac059.580

**Published:** 2022-12-20

**Authors:** Emily Willroth, Jing Luo, Eileen Graham, Mina Antic, Maria Lopes, Avron Spiro, Daniel Mroczek, Lewina Lee

**Affiliations:** Washington University in St. Louis, St. Louis, Missouri, United States; Northwestern University, Chicago, Illinois, United States; Northwestern University, Chicago, Illinois, United States; Massachusetts General Hospital and Harvard Medical School, Boston, Massachusetts, United States; Massachusetts General Hospital, Boston, Massachusetts, United States; Boston University, Jamaica Plain, Massachusetts, United States; Northwestern University, Chicago, Illinois, United States; Boston University School of Medicine, Boston, Massachusetts, United States

## Abstract

Exposure to stressors in childhood is theorized to sensitize individuals to stressors experienced later in life. One way that stress sensitization might manifest is through greater affective reactivity to daily stressors. In turn, greater affective reactivity to daily stressors has been associated with poorer health and increased mortality risk. The present pre-registered investigation tested greater affective reactivity to daily stressors in later life as a potential mediator of the association between early life stressors and mortality risk in a sample of 144 men from the VA Normative Aging Study. Partially consistent with our hypotheses, greater early life psychosocial stressors were associated with greater positive (but not negative) affective reactivity to daily stressors in later life. However, neither early life psychosocial stressors nor affective reactivity to daily stressors were significant predictors of mortality risk. We will discuss implications of these findings for theories of stress experience across the lifespan.

